# Extracting drug-enzyme relation from literature as evidence for drug drug interaction

**DOI:** 10.1186/s13326-016-0052-6

**Published:** 2016-03-07

**Authors:** Yaoyun Zhang, Heng-Yi Wu, Jingcheng Du, Jun Xu, Jingqi Wang, Cui Tao, Lang Li, Hua Xu

**Affiliations:** School of Biomedical Informatics, University of Texas Health Science Center at Houston, Houston, TX USA; School of Medicine, Indiana University, Indianapolis, IN USA

**Keywords:** Drug-enzyme interaction, Pharmacokinetic drug-drug interactions, Semantic graph kernel, Ontology-based inference, Relation extraction, Literature mining

## Abstract

**Background:**

Information about drug–drug interactions (DDIs) is crucial for computational applications such as pharmacovigilance and drug repurposing. However, existing sources of DDIs have the problems of low coverage, low accuracy and low agreement. One common type of DDIs is related to the mechanism of drug metabolism: a DDI relation may be caused by different interactions (e.g., substrate, inhibit) between drugs and enzymes in the drug metabolism process. Thus, information from drug enzyme interactions (DEIs) serves as important supportive evidence for DDIs. Further, potential DDIs present implicitly could be detected by inference and reasoning based on DEIs.

**Methods:**

In this article, we propose a hybrid approach to combining machine learning algorithm with trigger words and syntactic patterns, for DEI relation extraction from biomedical literature. The extracted DEI relations are used for reasoning to infer potential DDI relations, based on a defined drug-enzyme ontology incorporating biological knowledge.

**Results:**

Evaluation results demonstrate that the performance of DEI relation extraction is promising, with an F-measure of 84.97 % on the in vivo dataset and 65.58 % on the in vitro dataset. Further, the inferred DDIs achieved a precision of 83.19 % on the in vivo dataset and 70.94 % on the in vitro dataset, respectively. A further examination showed that the overlaps between our inferred DDIs and those present in DrugBank were 42.02 % on the in vivo dataset and 19.23 % on the in vitro dataset, respectively.

**Conclusions:**

This paper proposed an effective approach to extract DEI relations from biomedical literature. Potential DDIs not present in existing knowledge bases were then inferred based on the extracted DEIs, demonstrating the capability of the proposed approach to detect DDIs with scientific evidence for pharmacovigilance and drug repurposing applications.

## Background

Drug–drug interaction (DDI) is a situation when one drug alters the effect of another drug in a clinically meaningful way [1]. It has been demonstrated as one of the major causes of adverse drug reactions and a threat to public health [2–4]. Existing resources of DDIs include expert-curated knowledge bases such as DiDB (http://www.druginteractioninfo.org/), DrugBank (http:// www.drugbank.ca/), and pharmacy clinical support systems [5]. Significant efforts have been invested to incorporate DDIs into various data sources. However, existing sources suffer from the problems of low coverage [6], low accuracy [7] and low agreement [8].

Under such circumstance, scientific evidence revealing the mechanism behind the drug interactions are necessary to provide support for reliable DDI information [9]. One common type of DDIs is related to the mechanism of drug metabolism. For example, suppose drug A is a substrate of enzyme E, i.e., enzyme E is responsible for the metabolism of drug A. If the enzyme is inhibited or induced by drug B, the metabolism process of the drug A may be affected. Thus, the bioavailability of drug A could be different than expected, potentially causing adverse effect [10]. Therefore, drug-enzyme interactions (DEIs) serve as one type of important supportive evidence for DDIs. Besides, DDIs not explicitly stated in text may be detected by linking and reasoning over DEIs published in different scientific articles.

Since newly reported DEIs are rapidly accumulating in the huge archive of scientific literature [11], text mining techniques are needed to automatically extract DEIs as supportive scientific evidence for DDIs [6]. One pilot work in this direction is [10], which tried to extract the relations between drugs and enzymes based on properties of drug metabolism; potential DDIs were then detected by inference and reasoning. In [10], sentences in PubMed were stored as parse trees in a database, and SQL queries consisting of keywords and simple syntactic and semantic constraints were used to extract DEIs. SemRep [12], a widely used tool to extract relations from biomedical literature, also uses rule-based methods to extract DEI relations.

One problem with current DEI extraction methods is that their performance tend to be poor [10], given that sentences in scientific literature tend to be long and have complex structure. Hence, more data-driven, statistical methods such as machine learning algorithms are necessary to automatically improve the performance. Furthermore, no biological knowledge of concept hierarchies is involved in the inference process for DDIs currently. For example, if the drug Delavirdine is an inhibitor of CYP3A [13], it could be an inhibitor of all enzymes in the subfamily of CYP3A, such as CYP3A4. Potential DDIs between Delavirdine and drugs that are substrates of CYP3A4 could then be inferred. In this way, more implicit potential DDIs may be identified.

In this article, we propose a hybrid approach to extracting DEI relations. First, related drug enzyme pairs are extracted from sentences using the all-path graph kernel based machine-learning algorithm [14]. Specific DEI relation types are then assigned according to trigger words and syntactic patterns. After that, variations of drug and enzyme names are normalized to remove redundant relations. In the last step, inference rules are built based on the drug-enzyme ontology and biological knowledge about mechanisms of drug metabolism and interaction. Using these inference rules, the extracted DEI relations are then used for reasoning and inferring potential DDI relations.

Our approach differs from existing approaches in two ways. First, we propose a hybrid method to improve the performance of DEI relation extraction. Second, we establish an ontology-based inference process, incorporating hierarchical relations between enzymes. Our evaluation results using the DEI corpus [15] demonstrates that our proposed approach outperforms SemRep significantly. Moreover, implicit DDI relations are inferred with supportive evidence from DEIs, which may contribute to existing DDI knowledge bases such as DrugBank.

## Methods

Two DEI datasets, consisting of in vivo studies and in vitro studies, were used in this study. Our method involves three steps. First, related drug-enzyme pairs were extracted using an all-path graph kernel based machine-learning model. Different relation types were then assigned based on the trigger words and syntactic patterns. Second, variations of drug and enzyme names were normalized to remove redundant relations. In the last step, inference rules were built on the basis of drug-enzyme ontology and biological knowledge about mechanisms of drug metabolism and interaction. Using these inference rules, the extracted DEI relations were used for reasoning about potential DDI relations.

### Datasets

The corpus of DEI relations built by Wu, Karnik et al. [15] was employed in this study. The DEI relations were manually curated using 428 related abstracts from MedLine [15]. Related abstracts were retrieved from MedLine using the keywords of probe substrate/inhibitor/inducers for specific metabolism enzymes in queries. The abstracts for annotation were randomly selected from the search results. The abstracts in this corpus were categorized into two datasets for in vivo studies and in vitro studies, respectively, in order to accommodate the differences found between them the two study types. Two example sentences with DEI relations from the in vivo and in vitro studies are listed in Table 1.

**Table 1:** Example sentences with drug enzyme relations from literature

PMID	Study type	Sentence with drug enzyme interaction
10223773	in vivo	Rifampin (INN, rifampicin) is a potent inducer of CYP3A4 and some other CYP enzymes.
11353758	*in vitro*	Rifalazil-32-hydroxylation in microsomes was completely inhibited by CYP3A4-specific inhibitors (fluconazole, ketoconazole, miconazole, troleandomycin) and drugs metabolized by CYP3A4 such as cyclosporin A and clarithromycin, indicating that the enzyme responsible for the rifalazil-32-hydroxylation is CYP3A4.

All the drug enzyme pairs that co-occur in one sentence were considered as candidate DEI pairs. The interaction relations between drug pairs were labeled as “DEI” (positive) or “NDEI” (negative). Table 2 shows the statistics from the two datasets.

**Table 2:** Statistics of drug enzyme relation datasets

	Dataset	Abstract	Sentence	Relation pair	True pair
in vivo	Train	174	2114	1287	326
	Test	44	546	364	110
*in vitro*	Train	168	1894	4337	1360
	Test	42	475	1262	348

### Relation extraction

Our relation extraction method consisted of three steps. First, we represented sentences with dependency-based syntactic structures. Second, all-path graph kernels describing the syntactic connections within the sentences were generated from those representations. A Support Vector Machine (SVM) classifier was trained based on the graph kernels to generate a predictive model and to identify if the candidate drug-enzyme pair was related. In the last step, trigger words and syntactic patterns of different mechanisms of metabolism, i.e., “substrate”, “inhibitor”, “inducer”, were used for specific DEI relation assignment.

#### Sentence representation

Sentences with candidate DEI pairs were represented by the dependency syntactic structure. For generalization, specific drug/enzyme names in a candidate DEI pair were replaced with “*Drug*”/“*Enzyme*” in a preprocessing step. For example, CYP2C9 and sildenafil in *S*_*1*_ were replaced with *Enzyme1* and *Drug1*.

*Enzyme1*

*Drug1*

*S*_*1*_: CYP2C9 exhibited substantial sildenafil N-demethylase activity.

**Fig. 1:**
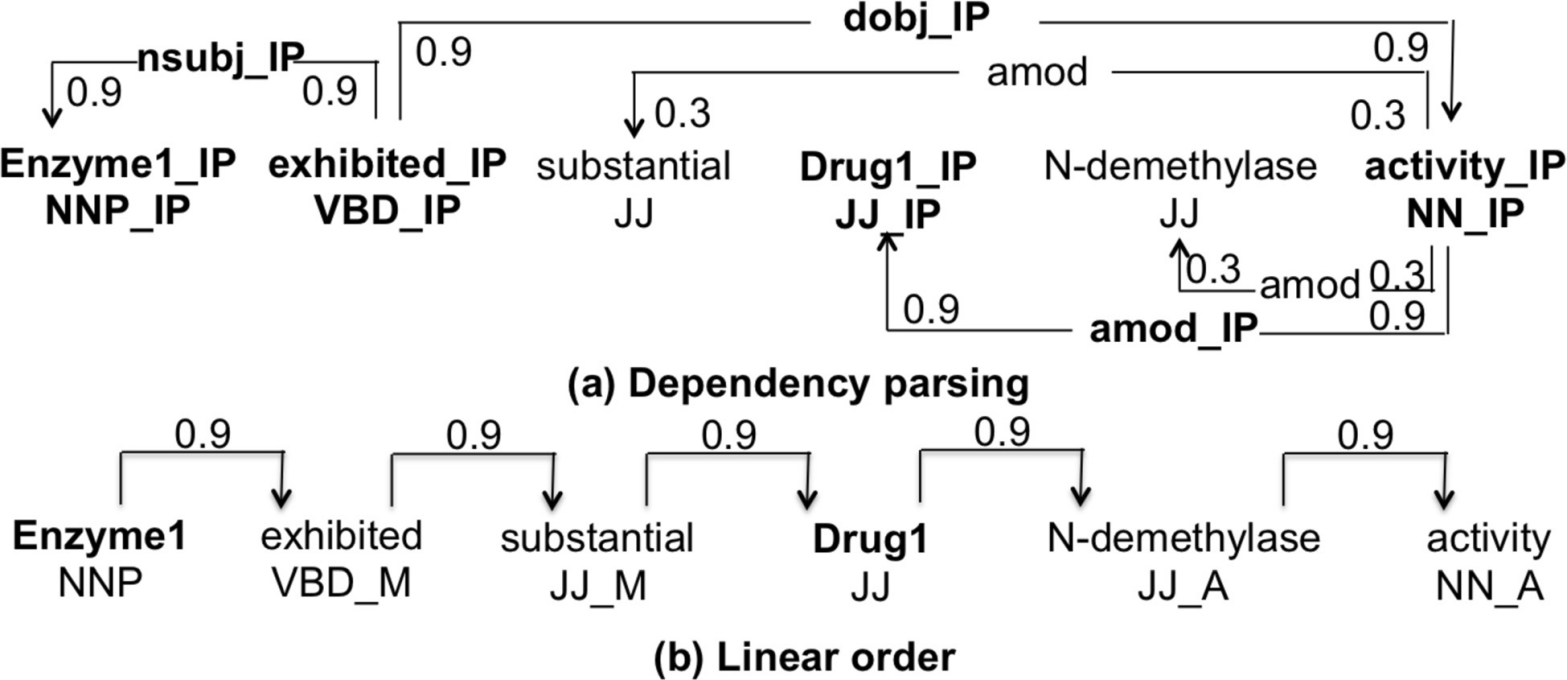
Illustration of the all-path graph representation. The candidate interaction pair is marked as “Enzyme1” and “Drug1”. The shortest path between the enzyme and the drug is shown in bold. In the dependency based sub-graph (**a**), all nodes in the shortest path are specialized using a post-tag (IP). In the linear order subgraph (**b**), possible tags are (B)efore, (M)iddle, and (A)fter

Dependency graph of a sentence was constructed based on its syntactic parse structure. It was a directed graph that included two types of vertices: a word vertex containing its lemma and part-of-speech tags (POS), and a dependency vertex containing the dependency relation between words. In addition, both types of vertices contained their positions, which differentiated them from other vertices. Figure 1(a) illustrates the dependency graph of *S*_*1*_. Since the words connecting the candidate entities in a syntactic representation are particularly likely to carry information regarding their relationship [16], the labels of the vertexes on the shortest undirected paths connecting “*drug*” and “*enzyme*” were differentiated from the labels outside the paths using a special tag “IP”. Further, the edges were assigned weights; all edges on the shortest paths received a weight of 0.9 and other edges received a weight of 0.3 as in [14]. Thus, the shortest path is emphasized while also considering the other words outside the path as potentially relevant.

#### All-path graph kernel

A graph kernel calculates the similarity between two input graphs by comparing the relations between common vertices. The weights of the relations are calculated using all possible paths between each pair of vertices. Our method follows the all-paths graph kernel proposed by Airola et al. [14]. The kernel represented the target pair using graph matrices based on two sub-graphs. The first sub-graph represented the structure of a sentence using the dependency graph; the second sub-graph represented the word sequence in the sentence, and each of its word vertices contained its lemma, its relative position to the target pair and its POS; all edges received a weight of 0.9 as in [14] (please see Fig. 1(b)).

Assuming that *V* represents the set of vertices in the graph, calculation of the similarity between two graphs used two types of matrices: edge adjacent matrix *A* and label matrix *L*. The graph is represented with the adjacent matrix *A* ∈ *R*^|*V*| × |*V*|^ whose rows and columns were indexed by the vertices, and [*A*]_*i,j*_ contains the weight of the edge connecting *v*_*i*_ ∈ *V* and *v*_*j*_ ∈ *V* if such an edge exists, and 0 otherwise. In addition, the labels were presented as a label allocation matrix *L* ∈ *R*^|*I*| × |*V*|^, so that *L*_*i,j*_ = 1 if the *j-th* vertex had the *i-th* label, and L_i,j_ = 0 otherwise. Using the Neumann Series, a graph matrix *G* is calculated as:

1 $$ G={L}^T{\displaystyle {\sum}_{n=1}^{\infty }{A}^nL={L}^T\left({\left(I-A\right)}^{-1}-I\right)L} $$

This matrix sums up the weights of all the paths between any pair of vertices, where each entry represents the strength of the relation between a pair of vertices. Given two instances of graph matrices *G*′ and *G*″, the graph kernel *K*(*G*^'^, *G*^' '^) is defined as follows:

2 $$ K\left({G}^{\hbox{'}},{G}^{\hbox{'}\hbox{'}}\right)={\displaystyle {\sum}_{i=1}^{\left|L\right|}{\displaystyle {\sum}_{j=1}^{\left|L\right|}{G}_{ij}^{\hbox{'}}{G}_{ij\hbox{'}}^{\hbox{'}\hbox{'}}}} $$

#### Relation type assignment

After recognizing the related drug-enzyme pairs, the rules generated from trigger words and common syntactic patterns of various mechanisms of drug metabolism were used to assign specific relations, i.e., “*isSubstrateOf*”, “*isInhibitorOf*” and “*isInducerOf*”. Some rules of each relation are illustrated in Table 3. For example, the sentence “*The metabolism of MDZ, which is specifically metabolized by CYP3A4 in humans*” matches the pattern of “Drug… metabolized by Enzyme”, from which the relation that MDZ is a substrate of CYP3A4 could be identified. The source code for relation assignment rules can be accessed following the link https://sbmi.uth.edu/ccb/resources/dei.htm.

**Table 3:** Trigger words and syntactic patterns of different DEI relation types

Relation	Trigger words & syntactic patterns
*isSubstrateOf*	Drug … *mediated/catalyzed/metabolized* by Enzyme Enzyme … *responsible for/contribute to* Drug *metabolism* *Metabolism…* Drug(Enzyme)
*isInhibitorOf*	Drug … an inhibitor of Enzyme Enzyme inhibitor (Drug) Enzyme inhibit Drug …activity
*isInducerOf*	Drug induced…Enzyme Drug … as a potent inducer of Enzyme

### Concept normalization

In the DEI datasets employed in this study, the drug names were recognized using DrugBank and regular expressions of various drug metabolites; enzyme names were recognized using regular expressions of various forms of enzymes [15]. Many variations of drugs and enzymes were annotated in the dataset. For example, “CBZ” is an abbreviation of the drug “Carbamazepine”. Both “P4503A4” and “3A4” were mentions of the enzyme “CYP3A4”. Hence, drug names and enzyme names were first normalized to reduce relation redundancy before the reasoning step. Drug names were normalized to concepts in Unified Medical Language System (UMLS) [17] using MetaMap [18]. Enzyme names were normalized to CYP450 enzymes, as defined in the human cytochrome P450 allele nomenclature database, http://www.cypalleles.ki.se/. The number of extracted DEIs were reduced accordingly.

### Knowledge representation and reasoning

#### Drug-enzyme *ontology definition*

To incorporate the knowledge of drug metabolism with the extracted DEI relations from biological literature, we created a DEI ontology. There are two classes in DEI ontology: *Drug* and *Enzyme*. Each extracted drug or enzyme was considered an individual of *Drug* or *Enzyme respectively*. Further, biological knowledge of mechanisms in drug metabolism were represented by object properties between *Drug* and *Enzyme* in the ontology. As shown in Table 4, five object properties were defined between *Drug* and *Enzyme*. We implemented the DEI ontology in OWL 2 (Web Ontology Language) [19]. OWL 2 uses description logic to represent formal semantics for semantic inference. OWL API (Application Programming Interface) was used for the creation and manipulation of the DEI Ontology [20].

**Table 4:** Logic facts definition for drug drug interaction inference

Drug enzyme relation	
* isSubstrateOf* (d, e)	Drug d is metabolized by enzyme e
* isInhibitorOf* (d, e)	Drug d inhibits the activity of enzyme e
* isInducerOf* (d, e)	Drug d induces the activity of enzyme e
Enzyme enzyme relation	
* isAncestorOf* (e1, e2)	Enzyme e1 is an ancestor of enzyme e2 in the enzyme family
Drug drug relation	
* DDI*(d1, d2)	Drug d1 and drug d2 have an interaction

#### Drug enzyme *ontology based inference*

After the ontology was populated, we defined property chain rules to infer new DDI. The following are three rules that we defined to infer DDI:

- Rule 1: *isSubstrateOf* (d1, e) and *isInhibitorOf* (d2, e) - > *DDI* (d1, d2)
- Rule 2: *isSubstrateOf* (d1, e) and *isInducerOf* (d2, e) - > *DDI* (d1, d2)
- Rule 3: *isSubstrateOf* (d1, e1) and *isAncestorOf* (e1, e2) - > *isSubstrateOf* (d1, e2)

Rule 1 and Rule 2 encode the knowledge that if a given drug d1 is a substrate of enzyme e, and drug d2 is an inhibitor/inducer of enzyme e, then drug d1 and d2 have a potential interaction. Rule 3 defines that the *isSubstrateOf* relation can be inherited by a descendant enzyme from its ancestors. Similar rules of inheritance were then defined for the other drug-enzyme relations based on the enzyme hierarchical relations. The reasoner HermiT was employed for DDI relation inference, which could check consistency of ontologies, compute the classification hierarchy, and explain inferences (Horrocks, et al., 2012). The ontology can be downloaded from https://sbmi.uth.edu/ontology/files/DEIOntology.owl.

### Experiments

#### Machine learning (ML) algorithm

SVM algorithms are the dominant ML methods (Segura-Bedmar et al., 2013) among the existing DDI systems. This study used the sparse version of RLS, also known as the least squares SVM, to learn the DEI prediction model based on the all-path graph kernel [14].

#### Experimental setup

POS-tags and dependency trees of the datasets were generated by Stanford parser [21]. We used the standard evaluation measures (Precision, Recall and F- measure) to evaluate the performance. We evaluated the performance of our system on each test dataset after training on the corresponding training dataset. Because our datasets were imbalanced with much more ‘NDEI’ relations then “DEI” relations, the same candidate drug-enzyme pair present in multiple instances may be classified as ‘DEI’ in one instance and as ‘NDEI’ in another. In this case, we treated this candidate DEI pair as a true ‘DEI’ pair to enhance the precision. Hence, the performance evaluation of relation extraction was carried out at the entity-level instead of the sentence level.

The following systematic analyses were conducted based on the experiments implemented in our study:

1. Comparison of DEI relation extraction performance between the all-path graph kernel based model (GraphKernel) with the model of java simple relation extraction (JSRE) [22]. JSRE is another state-of-the-art relation extraction model. It has demonstrated comparable performance with the all-path graph kernel based model in protein-protein interaction relation extraction [14, 23]. Different kernel options and parameters provided by JSRE were examined by 10-fold cross validation on the training datasets. The optimal performance of JSRE was used for comparison in our study, which was achieved by employing the shallow linguistic context kernel with default parameters. Further comparison was made with the existing knowledge base SemMedDB of literature relations, which was built using the SemRep system [12]. To select relations between drugs and genes from SemMedDB, PMIDs were used as one of the query constraints, to ensure that the selected relations were within the same publications as the test datasets.
2. Comparison of generated DDI relations with DrugBank: for each drug, we looked into the overlap between the generated DDI relations with the DrugBank. Specfically, novel DDI relations generated in our study were examined by checking their supportive evidence.

## Results and discussion

### Performance of drug-enzyme relation extraction

Table 5 illustrates the performance of DEI relation extraction. As can be seen, JSRE obtained higher recall on both datasets as compared to the GraphKernel (in vivo: 83.67 % vs. 85.30 %; in vitro: 57.96 % vs. 71.20 %), while its precision dropped significantly (in vivo: 86.32 % vs. 72.70 %; in vitro: 75.51 % vs. 61.90 %). Overall, GraphKernel outperformed JSRE on the in vivo dataset (*F*_*1*_: 84.97 % vs. 78.50 %), with a slightly lower *F*_*1*_ on the *in vitro* dataset (*F*_*1*_: 65.58 % vs. 66.20 %). The DEI relations extracted by SemRep are of only two types (‘INTERACTS_WITH’ and ‘INHIBITS’), most of which were of the ‘INTERACTS_WITH’ type (105/165). Therefore, only the performance with reference to recognition of related drug-enzyme pairs was compared between our method and SemRep. As shown in Table 5, GraphKernel outperformed SemRep significantly (in vivo: 84.97 % vs. 30.53 %; in vitro: 65.58 % vs. 15.32 %). Besides, the performance of the in vivo study dataset was much higher than that of the in vitro study dataset. Specifically, in the in vitro study dataset, the recall was much lower as compared to the in vivo study dataset (GraphKernel: 84.97 % vs. 65.58 %; SemRep: 30.53 % vs. 15.32 %).

**Table 5:** Drug enzyme relation extraction performance

Dataset	Method	*P*	*R*	*F*_*1*_
in vivo	GraphKernel	86.32 %	83.67 %	84.97 %
	JSRE	72.70 %	85.30 %	78.50 %
	SemRep	60.60 %	20.41 %	30.53 %
in vitro	GraphKernel	75.51 %	57.96 %	65.58 %
	JSRE	61.90 %	71.20 %	66.20 %
	SemRep	55.73 %	8.88 %	15.32 %

Table 6 illustrates the performance of our system in terms of drug-enzyme relation assignment. After drug and enzyme normalizations, 30 *isSubstrateOf*, 29 *isInhibitorOf* and 7 *isInducerOf* relations were identified in the in vivo dataset totally; 62 *isSubstrateOf*, 67 *isInhibitorOf* and 5 *isInducerOf* relations were identified in the in vitro dataset. As can be seen, the performance for the *isSubstrateOf* relation was relatively higher among the three relations in both datasets (in vivo: 87.48 %; in vitro: 72.79 %). The performance in the in vitro dataset is much lower than that in the in vivo dataset, since many of DEI pairs were already lost in the first stage of recognizing related drug-enzyme pairs (Table 5). The extracted relations were used to populate the DEI ontology defined in Section 2.4.1. Totally, the current ontology contains 104 individuals in *Drug*, 16 individuals in *Enzyme*, and 213 triples for drug metabolism, including 81 *isSubstrateOf* triples, 96 *isInhibitorOf* triples, 12 *isInducerOf* triples, and 24 *isAncestorOf* triples.

**Table 6:** Drug enzyme relation assignment performance

Dataset	Relation	*P*	*R*	*F*_*1*_
in vivo	*isSubstrateOf*	89.34 %	85.71 %	87.48 %
	*isInhibitorOf*	83.33 %	77.42 %	80.27 %
	*isInducerOf*	71.43 %	57.14 %	63.49 %
in vitro	*isSubstrateOf*	80.15 %	66.67 %	72.79 %
	*isInhibitorOf*	73.88 %	55.37 %	63.30 %
	*isInducerOf*	69.74 %	40.00 %	50.84 %

### Performance of drug-drug interaction inference

Evaluation results of inferred DDIs are listed in Table 7. Totally, 181 DDIs were inferred from the in vivo dataset, and 376 DDIs were inferred from the in vitro dataset, respectively. For comparison, only relations between drugs present in DrugBank were examined during evaluation. Totally, 31 drugs and 40 drugs in the in vivo and in vitro datasets, were present in DrugBank respectively. For the drugs present both in our corpus and DrugBank, totally 119 DDIs were inferred from the in vivo dataset, of which 69 DDIs were not included in DrugBank; 234 DDIs were inferred from the in vivo dataset, of which189 DDIs were not included in DrugBank. As illustrated in Table 7, the overlap between inferred DDIs in this study and DrugBank was low (in vivo: 42.02 %; in vitro: 19.23 %). However, by manually checking the supportive evidences, i.e., the underlying DEI relations for those DDIs, it was verified that the inferred DDIs achieved a precision of 83.19 % for the in vivo dataset and 70.94 % for the in vitro dataset, respectively.

**Table 7:** Performance of drug drug relation inference

Dataset	*P*	*R*	*F*_*1*_	*DrugBankOverlap*
in vivo	83.19 %	52.84 %	64.63 %	42.02 %
in vitro	70.94 %	42.11 %	52.85 %	19.23 %

### Discussion

DEIs are important supportive evidence for DDIs. This study applied a hybrid approach for DEI relation extraction from biomedical literature. Reasoning was then conducted on the extracted DEIs to infer potential DDI relations, by incorporating biological knowledge into drug-enzyme ontology. Evaluation results demonstrated the effectiveness of our approach: potential DDIs were inferred with reliable precisions (in vivo: 80.30 %; in vitro: 72.09 %), indicating its capability to detect DDIs with scientific evidence.

The model of GraphKernel obtained much higher precision and lower recall than JSRE (Table 5). This demonstrated that GraphKernel and JSRE have advantages of different aspects on the DEI datasets. One potential explanation could be the essential kernel difference between these two models. JSRE only relies on shallow linguistic features of text, such as tokens, POS and lemmas, while GraphKernel combines shallow linguistic features with more complex structural syntactic features. Thus, the constraints of JSRE were relatively relaxed on the text in comparison with GraphKernel, leading to the high recall of JSRE and the higher precision of GraphKernel. Overall, GraphKernel outperformed JSRE significantly on the in vivo dataset (*F*_*1*_: 84.97 % vs. 78.50 %), with a slightly lower *F*_*1*_ on the in vitro dataset (*F*_*1*_: 65.58 % vs. 66.20 %). This indicates that there is room for further improvement in the relation extraction from the in vitro dataset.

As shown in Table 5, our approach outperformed SemRep significantly in terms of DEI relation extraction. One possible reason could be that SemRep is a general information extraction tool for biomedical literature, which is not focused on the DEI relation. On the other hand, our model was trained on the datasets dedicated to DEI relations. Another possible reason is that instead of using rule-based methods as in SemRep, our study applied statistical machine-learning model first to recognize related drug-enzyme pairs to remove false positive DEI relation pairs and to improve the performance. As an illustration, in the sentence “*the possibility of in vivo drug interaction of azelastine and other drugs that are mainly metabolized by CYP2D6*”, the candidate relation pair of azelastine and CYP2D6 matches the pattern of the *isSubstrateOf* relation. However, it is a false positive relation and is removed in the first step by the statistical model.

Although for the drugs present both in our corpus and DrugBank, only 42.02 % of inferred DDIs from the in vivo dataset and 19.23 % from the in vitro dataset are covered by DrugBank, manual examination demonstrated that our approach could find potential DDI relations with supportive evidence. For example, from the sentence “*… and is probably caused by inhibition of CYP3A4 -mediated voriconazole metabolism*” (PMID: 16890574), we identified that the drug voriconazole is a substrate of CYP3A4; meanwhile, from the sentence “*…oxcarbazepine (OXCZ) are well-known inducers of drug metabolism via CYP3A4*” (PMID: 17346248), we identified the relation that the drug oxcarbazepine is an inducer of CYP3A4. One potential interaction between voriconazole and oxcarbazepine could then be inferred, which is not listed in DrugBank. More examples of inferred DDIs as well as their supportive evidence from literature are listed in Table 8.

**Table 8:** Examples of inferred drug drug interactions and supportive evidence from literature

Drugs with interaction	Enzyme	Evidence
Carbamazepine/oxcarbazepine quinidine	CYP3A4	We performed a study in healthy volunteers to investigate the relative inductive effect of CBZ and OXCZ on CYP3A4 activity using the metabolism of quinidine as a biomarker reaction…We confirm a clinically significant inductive effect of both OXCZ and CBZ. (PMID: 17346248)
Lidocaine fluvoxamine	CYP1A2	Lidocaine is metabolized by cytochrome P450 3A4 (CYP3A4) and CYP1A2 enzymes…We conclude that inhibition of CYP1A2 by fluvoxamine considerably reduces the presystemic metabolism of oral lidocaine… (PMID: 16918719)
Quinidine itraconazole	CYP3A4	Quinidine is eliminated mainly by CYP3A4-mediated metabolism… Itraconazole increases plasma concentrations of oral quinidine, probably by inhibiting the CYP3A4 isozyme during the first-pass and elimination phases of quinidine. (PMID: 9390107)
Propofol orphenadrine	CYP2B6	Involvement of human liver cytochrome P4502B6 in the metabolism of propofol… orphenadrine, a CYP2B6 inhibitor, reduced the rate constant of propofol by liver microsomes by 38 % (*P* < 0.05)… (PMID: 11298076)
Rifalazil fluconazole	CYP3A4	Rifalazil-32-hydroxylation in microsomes was completely inhibited by CYP3A4-specific inhibitors (fluconazole, …) … indicating that the enzyme responsible for the rifalazil-32-hydroxylation is CYP3A4. (PMID: 10923859)

Despite the fact that our proposed method of DEI relation extraction achieved a *F*_*1*_ of 84.97 % on the in vivo dataset, the *F*_*1*_ of 65.58 % obtained on the in vitro dataset is still low. Based on our empirical observation, the major reason for the performance difference between these two datasets lied in the essential difference of their linguistic structures, which originated from the difference between the in vivo and in vitro studies. In vivo studies focus on evaluating the effect of an investigational drug on other drugs, by checking the changes of pharmacokinetic parameters. Different from in vivo studies, in vitro studies can qualitatively provide the mechanisms of a potential DDI based on the observation of enzyme kinetics parameters. Thus, sentences in the in vitro dataset contained more drug enzyme interactions; whereas they were also much complex than those in the in vivo dataset, with more multiple clauses, long conjunctive structures and rare patterns. When we looked into the errors of DEI relation extraction, especially in the *in vitro* dataset, we found that the major causes of false negative instances include conjunctive structures of drugs/enzymes (e.g., “Studies using the ***CYP3A4*** inhibitors ketoconazole, ***troleandomycin***, and ***erythromycin***”), and the rare patterns uncovered by the statistical model (e.g. “Induction of ***CYP2C9*** would explain the increased systemic elimination of ***glipizide***”). On the other hand, the major causes of false positive instances include the inability to catch the context information differentiating between positive and negative relations (e.g., the word “confirm” indicates the uncertainty of the DEI relation in the sentence “… to ***confirm*** that fluvoxamine inhibits CYP2C19”), and wrong predictions between drugs and enzymes across multiple clauses, as in the sentence “Greater inhibition was produced by the less selective CYP3A inhibitors ***parathion***, ***quinidine***, and ***ketoconazole***; ***CYP1A*** inhibitors were ineffective.”.

The above problems should be addressed in the future to further improve the DEI relation extraction performance. Specifically, additional advanced methods tailored to the *in vitro* dataset should be explored, including automatic pattern recognition methods to identify conjunctive structures of drugs/enzymes, multiple clauses split before feature extraction, keyword expansion to indicate the uncertainty (e.g., “to determine” and “was examined”).

One limitation of our current work is the size of the annotated corpus. For practical usage, we plan to apply our system to all the related articles in PubMed to obtain a more comprehensive list of DEIs and potential DDIs. Besides, further improvements of our system may need to be conducted after evaluation on a larger DEI corpus. In addition to narrative literature text describing DEIs, tables of DEIs with details of interactions in the published full text articles are another valuable resource to obtain such information that we plan to incorporate. Extracting DEIs from tables is more straightforward and potentially have more accurate results as compared to the text. However, in comparison to accessing titles and abstracts of articles through MedLine, one problem of tables is that the automatic access to full text is limited. Actually, these two resources could be complementary to each other for mining DEIs from biomedical literature. In our future work, methods of mining tables from DEI related articles would be explored. Another drawback of our current approach for DDI relation inference is that the information of specific conditions required for the occurrence of DEIs and DDIs, such as dosages of drugs, was not considered. Information of such conditions is also very critical for supportive evidence for DDI relations, which should be taken into consideration in the next step.

## Conclusion

Our study proposes a hybrid approach of combining machine-learning algorithm with rule-based patterns to extract DEIs from biomedical literature, from which potential DDI relations can be inferred by reasoning. Evaluation results demonstrate that the performance of DEI relation extraction outperformed SemRep significantly, with a F-measure of 84.97 % on the in vivo dataset and 65.58 % on the *in vitro* dataset. Moreover, potential DDIs not present in DrugBank were also inferred, indicating that this proposed approach could be used to detect DDIs supported by scientific evidence of drug metabolism and interaction.
