# Erratum to: Extracting drug-enzyme relation from literature as evidence for drug drug interaction

**DOI:** 10.1186/s13326-016-0063-3

**Published:** 2016-04-19

**Authors:** Yaoyun Zhang, Heng-Yi Wu, Jingcheng Du, Jun Xu, Jingqi Wang, Cui Tao, Lang Li, Hua Xu

**Affiliations:** School of Biomedical Informatics, University of Texas Health Science Center at Houston, Houston, TX USA; School of Medicine, Indiana University, Indianapolis, IN USA

Following the publication of the original article [1] it was brought to our attention that an important acknowledgement from co-author Jingcheng Du was missing from the manuscript. The authors would like to apologise for this oversight and now take the opportunity to gratefully acknowledge the support from the UTHealth Innovation for Cancer Prevention Research Training Program Pre-doctoral Fellowship (Cancer Prevention and Research Institute of Texas grant # RP140103).
